# Global changes in nitration levels and DNA binding profile of *Trypanosoma cruzi* histones induced by incubation with host extracellular matrix

**DOI:** 10.1371/journal.pntd.0008262

**Published:** 2020-05-29

**Authors:** Rubens Daniel Miserani Magalhães, Eliciane Cevolani Mattos, Andrei Rozanski, Pedro Alexandre Favoretto Galante, Giuseppe Palmisano, Angela Kaysel Cruz, Walter Colli, Anamaria Aranha Camargo, Maria Júlia Manso Alves

**Affiliations:** 1 Departamento de Bioquímica Instituto de Química, Universidade de São Paulo, São Paulo, São Paulo, Brazil; 2 Centro de Oncologia Molecular, Hospital Sírio Libanês, São Paulo, Brazil; 3 Departamento de Biologia Celular e Molecular, Faculdade de Medicina de Ribeirão Preto, Universidade de São Paulo, Ribeirão Preto, SP, Brazil; 4 Departamento de Parasitologia, Instituto de Ciências Biomédicas, Universidade de São Paulo, São Paulo, Brazil; Yale University School of Medicine, UNITED STATES

## Abstract

Adhesion of *T*. *cruzi* trypomastigotes to components of the extracellular matrix (ECM) is an important step in mammalian host cell invasion. We have recently described a significant increase in the tyrosine nitration levels of histones H2A and H4 when trypomastigotes are incubated with components of the ECM. In this work, we used chromatin immunoprecipitation (ChIP) with an anti-nitrotyrosine antibody followed by mass spectrometry to identify nitrated DNA binding proteins in *T*. *cruzi* and to detect alterations in nitration levels induced upon parasite incubation with the ECM. Histone H1, H2B, H2A and H3 were detected among the 9 most abundant nitrated DNA binding proteins using this proteomic approach. One nitrated tyrosine residue (Y29) was identified in Histone H2B in the MS/MS spectrum. In addition, we observed a significant increase in the nitration levels of histones H1, H2B, H2A and H4 upon parasite incubation with ECM. Finally, we used ChIP-Seq to map global changes in the DNA binding profile of nitrated proteins. We observed a significant change in the binding pattern of nitrated proteins to DNA after parasite incubation with ECM. This work provides the first global profile of nitrated DNA binding proteins in *T*. *cruzi* and additional evidence for modification in the nitration profile of histones upon parasite incubation with ECM. Our data also indicate that the parasite interaction with the ECM induces alterations in chromatin structure, possibly affecting nuclear functions.

## Author summary

*T*. *cruzi*, the etiological agent of Chagas’ disease, interacts with ECM in order to invade cells and establish the infection in the mammalian host. Studying this interaction, our group has recently described changes in the nitration profile of histones upon incubation of the parasite with ECM. Herein we used a proteomic approach to identify nitrated DNA binding proteins in *T*. *cruzi*. We also used ChIP-Seq to investigate whether the contact of the parasite with host ECM is capable of promoting changes in the DNA binding pattern of nitrated proteins. We observed significant alterations in the nitration levels and DNA binding profile of *T*. *cruzi* histones upon parasite incubation with ECM.

## Introduction

*Trypanosoma cruzi*, the causative agent of Chagas’ disease, completes its life cycle alternating between invertebrate (triatomine insects) and vertebrate (mammals, including humans) hosts. To survive in different hosts *T*. *cruzi* undergoes marked morphological and functional changes [1,2]. The signaling mechanisms that lead this parasite to adapt to these environmental changes are beginning to be understood.

Adhesion of *T*. *cruzi* trypomastigotes to components of the extracellular matrix (ECM) is an important step in mammalian host cell invasion [3,4]. We have recently reported that nitric oxide (NO) signaling is down-regulated when *T*. *cruzi* trypomastigotes are incubated with ECM. Inhibition of NOS activity, with the expected decrease in NO production, as well as decrease in cGMP concentration, was observed when trypomastigotes are incubated with ECM. In addition, we observed that parasite adhesion to ECM significantly modulates NO-mediated protein post-translational modifications (PTMs). Interestingly, in spite of a global down regulation of protein S-nitrosylation and nitration, a significant increase in the tyrosine nitration levels of histones H2A and H4 was observed upon incubation of the parasites with the ECM [4], pointing out to the specificity of the modification.

Although histones are less conserved in trypanosomatids than in other eukaryotes [5], trypanosomatids appear to have a chromatin structure and organization similar to other eukaryotes, with the DNA organized into nucleosomes having canonical (H2A, H2B, H3, H4) and variant (H2A.Z, H2B.V, H3.V) histones and a H1 histone associated to the linker DNA [6–9]. The presence of PTMs in histones, including acetylation, methylation, and phosphorylation, as well as unusual PTMs, such as alternative lysine acylation, glutarylation, crotonylation, malonylation, succinylation and N-terminal methylation, have been reported recently in trypanosomatids [10–14]. Considering a repertoire of 13 distinct chemical modifications, the number of modified sites and the many residues, which are substrates of different modifications, more than 176 PTMs were described by proteomic analysis in *T*. *cruzi* histones (also referred to as histone code) [12]. The description of nitrated tyrosines in *T*. *cruzi* histones [4] adds to this number.

Originally considered only as an indication of cellular stress, nitration of tyrosine residues in proteins is currently viewed as a PTM with an important signaling function [15–18]. In this context, the increase in tyrosine nitration levels of histones H2A and H4 observed upon incubation of the parasite with the ECM suggests a possible role of NO in the regulation of chromatin structure and in the control of nuclear functions in *T*. *cruzi* during host cell invasion [4]. In the present work, we used chromatin immunoprecipitation (ChIP) with an anti-nitrotyrosine antibody, followed by mass spectrometry to characterize alterations in tyrosine nitration levels of DNA binding proteins in *T*. *cruzi* induced by the parasite interaction with the ECM. We also used ChIP-seq to map global changes in DNA binding sites of nitrated proteins after parasite incubation with ECM. This work provides the first global profile of *T*. *cruzi* nitrated DNA binding proteins and, most importantly, provides evidence for modification in the nitration profile of *T*. *cruzi* histones upon parasite incubation with ECM. Our data also indicate that incubation of the parasite with the ECM induces alterations in the interaction pattern of nitrated histones with DNA, possibly affecting nuclear functions.

## Methods

### *T*. *cruzi* trypomastigotes cultivation and incubation with ECM

Cell culture of *T*. *cruzi* trypomastigotes (Y strain) were maintained by infection of LLC-MK_2_ cells, as described [19] in Minimum Essential Medium (MEM–GIBCO) containing 2% of Fetal Bovine Serum (FBS), under 5% CO_2_ and 37°C. Five days after infection of the host cell, culture supernatants containing the trypomastigotes were centrifuged and purified as described [20]. The commercially available extracellular matrix (Geltrex LDEV-Free Reduced Growth Factor Basement Membrane Matrix, Gibco, 150 μl ECM per sample) was used to incubate part of the trypomastigotes (5 x 10^8^ cells in 5 ml MEM) as described [21]. After incubation, the parasites were collected and used for chromatin extraction (sample named MTy). As a control, 5 x 10^8^ trypomastigotes from the same culture were subjected to the same conditions mentioned above in the absence of ECM (sample named Ty). The samples were frozen at -80°C for further use.

### Chromatin immunoprecipitation

Trypomastigotes (5 x 10^8^ cells) from MTy and Ty samples were lysed and the proteins of interest binding to chromatin were immunoprecipitated according to Lee et al., 2006 [22], with modifications (Detailed methodology is presented in S1 Text).

### Mass spectrometry analysis

For mass spectrometry analysis, 2 x 10^8^ cells of *T*. *cruzi* trypomastigotes from one of the replicates used in Chip-seq analysis (Rep 1) were treated with 0.3% formaldehyde, lysed in three steps and the proteins immunoprecipitated as described above for ChIP-seq protocol. One of the fractions of Ty-Rep1 was immunoprecipitated with IgG (mouse) to be used as control. After the reverse-crosslink step, the proteins were precipitated by acetone, resuspended in 100 mM sodium bicarbonate buffer, 8 M urea and frozen at -80°C until the digestion step. Immunocaptured proteins were treated with 10 mM DTT in 50 mM ammonium bicarbonate (Ambic) for 45 min at 30°C to reduce disulfide bridges. The reduced cysteines were alkylated with 40 mM iodoacetamide for 30 min in the dark. The samples were diluted 10 times using 50 mM Ambic. Proteins were digested with sequencing grade Trypsin (Promega, Madison, WI, USA) at 1:50 enzyme to substrate ratio and incubated overnight at 30°C. The enzymatic reaction was stopped by adding trifluoroacetic acid (TFA) to 1% final concentration. The peptides were desalted using Stage Tips with C18 disks as previously reported [23]. Peptide samples were resuspended in 0.1% TFA and analyzed using a nano-flow EASY-nLC 1200 system (Thermo Scientific) coupled to Orbitrap Fusion Tribrid mass spectrometer (Thermo Scientific). The peptides were loaded on Reprosil-Pur C18-AQ (3 μm) column and separated in an organic solvent gradient from 100% phase A (0.1% TFA) to 34% phase B (0.1% TFA, 95% ACN) during 60 min for a total gradient of 74 min at a constant flow rate of 250 nL/min. The full scan was acquired in the Orbitrap at a resolution of 120,000 FWHM in the 375–1600 m/z mass range with max injection time of 50ms and AGC target of 5e5. Peptide ions were selected using the quadrupole with an isolation window of 1.2 and fragmented with HCD MS/MS using a normalized collision energy of 30. Data dependent acquisition with a cycle time of 3 seconds was used to select the precursor ions for fragmentation. All raw data were accessed in Xcalibur software (Thermo Scientific). The RAW files were processed using the Maxquant software (version 1.5.3.8) for the protein quantification and identification [24]. Fasta file containing the *T*. *cruzi* protein sequences downloaded from UniProtKB (140068 sequences) plus common contaminants was used as database for peptide and protein identification. Protein quantification was performed using the label-free quantification algorithm implemented in the MaxQuant computational platform. The intensity-based absolute quantification (iBAQ) was calculated. Trypsin was included as cleavage enzyme with a maximum of two missed cleavages. Fixed modification was carbamidomethylation of cysteine and variable modifications were oxidation of methionine, tyrosine nitration and N-terminal protein acetylation. Identification with FDR<1% and no match to reverse database was considered for further analyses. The protein sequences of histones H2B were aligned using cluster omega web tool [25]. Seventeen sequences were recovered from Uniprot using the term “Histone H2B” in ‘Protein name [DE]’ field and the term “*Trypanosoma cruzi*” in ‘Organism[OS]’ field. The image of the alignment was created by Jalview tool [26].

### ChIP-Seq

The libraries of the immunoprecipitated DNA fragments from each sample and the corresponding INPUTs were constructed using the TruSeq ChIP Sample Prep Kit (Illumina), following the manufacturer's recommendations. Ten ng of purified DNA and 16 cycles were used in the amplification step. Libraries were quantified using KAPA library quantification kit (Kapa Biosystems). Sequencing of the immunoprecipitated and INPUT DNA libraries of the Ty and MTy samples was performed using the Nextseq 500 (Illumina). The paired-end fragment method was chosen in order to improve the mapping of low complexity regions in the *T*. *cruzi* genome. A pipeline was implemented for *in silico* analysis of two replicates using the BWA v0.7.15–1140 tool [27] to map the sequences; MACS v2.2 [28] to find genomic regions enriched by immunoprecipitation; DiffBind v2.2.12 [29] for normalization and detection of differentially represented genomic regions in Ty and MTy samples. S1 Fig shows the flowchart of the multi-replicate pipeline developed in each step and tools. Up to the differential binding analysis of ChIP-Seq peak data, both replicates were treated independently. Great care was taken in using parameters and stringent Phred quality score Q30 filter in order to generate accurate information. A pipeline was developed to identify broad enriched regions (Broad), generally associated with epigenetic control, and narrow enriched regions (Narrow), which can be recognized by transcription factors. The mapping of the reads for paired-end files (R1 and R2) of the replicates (Rep1 and Rep2) was done independently using "bwa aln". Bwa SAMPE was used to group the files containing the pairs of reads and Samtools [30] was used to obtain the final file properly filled in BAM format. At this point, a Phred mapping quality filter (MAPQ) of 30 was implemented to select the uniquely mapped reads in the *T*. *cruzi* genome. *T*. *cruzi* CL-Brener reference strain has genomic information from two haplotypes stored in the TriTrypDB (release 9) within the 41 chromosomes predicted for the parasite (*T*. *cruzi* CL Brener Esmeraldo-like (Esm-like), *T*. *cruzi* CL Brener Non Esm-like (NEsm-like) and contigs that are not assembled [31]. *T*. *cruzi* CL-Brener Esm-like haplotype, estimated at approximately 32 Mb, was used as reference genome, because at the time of our study, there was no genome assembled and annotated for the Y strain, which belongs to the same phylogenetic group of Esmeraldo (DTU I) [32,33]. The generated sequences provided approximately 140 X genome coverage. The reads mapped preferentially in *T*. *cruzi* CL Brener Esm-like haplotype (as quantified in a pilot experiment, S2 Fig). The search for enriched regions was done using two sets of MACS2 parameters since the specific antibody for the nitrous residue can recognize proteins with broader or narrower DNA binding patterns, named Broad or Narrow, respectively. Starting with the peak defined by the Narrow method, the binding region may be extended up to 4 times in each direction using the Broad method. In addition, the latter decreases the thresholds for detection of enriched peaks as compared to INPUT [28,34]. Duplicated reads were removed by the parameter “–keep-dup auto” using the default cutoff value of 1e-5 [34]. The GEM-Mappability Calculator [35] was used to calculate the effective genome size (EGS). MACS2 uses the EGS to set the detection threshold of candidates for regions enriched by immunoprecipitation (parameter λBG) [34]. The EGS of the *T*. *cruzi* CL-Brener Esm-like haplotype, originally composed of 32,529,070 bp, was 17,941,408 bp (55.15% of the original total size), as expected, given the high percentage of repetitive sequences present in the genome of *T*. *cruzi*. The DiffBind R package [29] was used to determine the differential profiles of enrichment in each treatment, Ty and MTy considering "False Discovery Rate" (FDR) lower than 5%. This tool considers the position of enriched regions defined in “Peak Calling” step, normalizing and quantifying the reads over these coordinates, even if the enriched region was found only in one treatment. The statistical analysis is done using DESeq 2 model [36].

### Search for proximal genes and functional analysis

The search for proximal genes was done using the ChIPpeakAnno tool [37]. Information about the parasite genes was obtained from the gff file (release 9.0) downloaded from the TriTrypDB database for the *T*. *cruzi* CL-Brener Esm-like genome (http://tritrypdb.org/common/downloads/release-9.0/TcruziCLBrenerEsmeraldo-like/gff/data/).

### ChIP-Seq quality metrics

The calculation of the post-mapping metrics was done using SPP tools [38,39] to obtain the cross-correlation metrics (Normalized Strand Cross-correlation (NSC) and Relative Strand Cross-correlation (RSC) coefficients), and the mel-ngs tool (https://github.com/mel-astar/mel-ngs/tree/master/mel-chipseq/chipseq-metrics) to calculate FRiP (Fraction of Reads in Peaks). The cross-correlation metric was calculated by a Pearson linear correlation between the Crick and the Watson strands [40]. Cross-correlation analysis between strands is based on the high partial overlap of reads in regions where the protein of interest binds to DNA obtained in high quality ChIP-seq experiments. ENCODE recommendation values for NSC, RSC and FRiP are >1.05 > 0.8 and >0.01 [40].

### Prediction of Strand Switch Regions (SSRs) coordinates

A homemade script was implemented in Python (v. 2.7.15) and Shell script to predict the SSRs coordinates. The script uses the annotated Protein Coding Sequences (CDSs) coordinates from the GFF file (TriTrypDB, version 37). The script assembles the genes from the same putative polycistronic transcriptional unit (PTU) delimiting convergent or divergent PTUs and defining Strand Switch Regions (SSR).

### Data visualization

The visualization of the enriched regions in the *T*. *cruzi* CL-Brener Esm-like genome was done using the IGV program [41]. Wig, bedGraph, bed, FASTA and GFF files were loaded into the program to visualize the enriched regions identified and close features of these regions in the parasite genome. The genomation packages [42] and ChIPseeker [43] were also used to visualize the enrichment and disposition of the enriched regions of interest in the parasite genome.

### Quantitative real time PCR (qPCR)

qPCR was used for validation of the differentially enriched regions identified after chromatin immunoprecipitation of the Ty and MTy samples by *in silico* analysis. An absolute quantification technique was used by standard curve with four points, converting the Ct values obtained to the estimated number of copies of the parasite genome. By analogy with the human genome the mass of the *T*. *cruzi* CL Brener Esm-like haplotype genome was defined as 0.064 pg. Four points referring to 100 (6.4 pg), 1000 (64 pg), 10000 (0.64 ng), 100000 (6.4 ng) copies of the genome were used on the curve. Genomic DNA extraction was performed using the commercial QIAamp DNA Mini kit (QIAGEN) following the manufacturer's recommendations. Quantitation of DNA from each sample was done using Qubit Fluorimetric Quantification (Qubit dsDNA HS Assay Kit, Thermo Scientific). The equipment used for this analysis was the 7900 Real-Time PCR System (Applied Biosystems) and SYBR Green PCR Master Mix reagent, following manufacturer recommendations for reaction volume (20 μL). The temperatures for amplification were 95°C, 10 min; 40 cycles (95°C, 15 sec; 60°C, 1 min). 0.3 μl of immunoprecipitated DNA from the Ty and MTy samples (replicates 1 and 2) and 200 nM primers were used as template (S1 Table). Pairs of primers were made for the amplification of 10 enriched regions (five of Ty and five of MTy) identified by the pipeline using Narrow MACSauto parameters, FDR 5% and with FC > 2. The primer3web program (version 4.0.0) [44] was used to design the primers, limiting the amplified regions from 100 to 150 bp and melting temperature (TM) from 55 to 58°C.

## Results

### Antibody characterization and identification of DNA binding nitrated proteins

The immunoprecipitated proteins from the enriched nuclear fraction of *T*. *cruzi* trypomastigotes using the anti-nitrotyrosine antibody were analyzed by bottom up proteomic approach. A total of 692 proteins were identified combining the three experimental conditions, of which 507 proteins were identified with at least two peptides (IgG, Ty and MTy) (S2 Table). Considering the proteins with annotated subcellular location, 48% are assigned to the nucleus.

**Fig 1:**
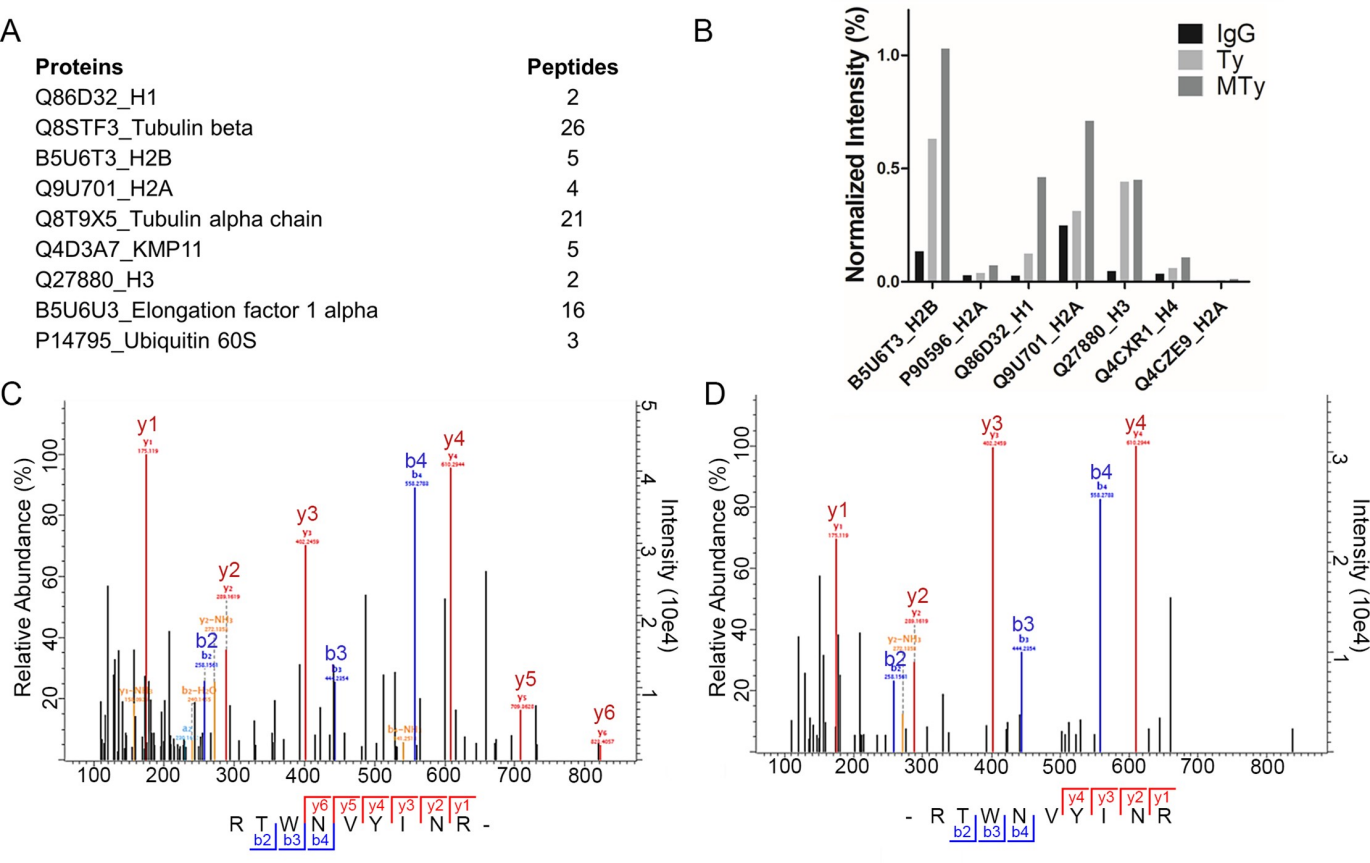
*T*. *cruzi* histones identified and quantified by immunoprecipitation with the anti-nitrotyrosine antibody. A–The nine most abundant proteins detected in both Ty and MTy conditions. B–Normalized intensity (%) of histones in the three conditions, IgG (control), Ty and MTy. C and D–Annotated MS/MS spectra of the RTWNVY(ni)INR peptide belonging to the histone H2B identified respectively in Ty and MTy samples. (ni) indicates the nitration site.

Using the normalized intensity-based absolute quantification, 4 out of the 9 most abundant proteins immunoprecipitated with anti-nitrotyrosine antibody in the Ty and MTy conditions were the histone subtypes H1, H2B, H2A and H3 (Fig 1A). Histone H4 was also identified among the 20 most abundant proteins. Comparison of the normalized intensity values of histone proteins among the IgG, Ty and MTy conditions showed higher level of histones immunoprecipitated in the MTy samples as compared to Ty, whereas levels were negligible in the IgG control (Fig 1B).

Analysis of the MS/MS data allowed the identification of nitrated histone H2B (B5U6T3). The RTWNVYINR peptide was nitrated at the Y29 residue. This peptide was identified in the Ty and MTy samples, but not in the IgG negative control. The annotated MS/MS spectra that have been identified in the Ty and MTy conditions are reported in Fig 1C and 1D, respectively. Y29, flanked by hydrophobic amino acids (…RTWNVYI… or…RWDLYI…), is conserved in all H2B aligned isoforms of *T*. *cruzi* except in the three truncated genes (S3 Fig).

In addition to histones, β-tubulin, microtubule-associated protein, basal body component, and proteins involved in nuclear RNA export, transcription, translation, biogenesis of ribosomes, stress response, among others, were identified also in the proteomic analysis of the immunoprecipitated material (S2 Table). Similar results have been previously described for the nuclear proteome [45].

### Identification of chromatin–bound nitrated proteins by ChIP-seq analysis

Since protein-DNA interactions regulate essential nuclear functions, ChIP-seq analysis was performed to map DNA regions associated with proteins able to be immunoprecipitated by anti-nitrotyrosine antibodies. Sequencing of the sample replicates was carried out using the Illumina sequencing platform (Table 1). It should be emphasized, that in spite of some differences, all samples remained at an average of exceptional quality above Q30 (S4 Fig).

**Table 1:** Profile of sequenced reads mapped to the reference genome.

Samples		Reads Mapped in *T*. *cruzi* CL Brener Esm-like Genome				
**Replicate 1**	**Total**	**Mapped**	**%Map**	**Q30_Filter**	**Q30_pMap**	**%After_Filter**
Ty	40,482,132	31,266,382	77	15,602,74	14,809,378	47
MTy	42,433,990	32,960,669	78	16,237,817	15,446,220	47
TyI	61,424,618	46,187,618	75	22,968,670	21,725,461	47
MTyI	87,585,574	63,839,771	73	32,044,018	30,358,694	48
**Replicate 2**	**Total**	**Mapped**	**%Map**	**Q30_Filter**	**Q30_pMap**	**%After_Filter**
Ty	37,939,186	29,464,323	78	14,916,522	14,156,699	48
MTy	33,785,266	26,682,664	79	13,471,040	12,785,893	48
TyI	135,289,894	85,230,876	63	41,403,070	39,013,742	46
MTyI	63,295,384	36,673,979	58	18,843,799	17,609,115	48

The sequenced reads were mapped against the *T*. *cruzi* CL-Brener Esm-like reference genome using the BWA program. Minimal mapping value observed was 58%, most of them were above 70% (Table 1). After application of quality filters, removal of reads from all the samples were proportional (Table 1, %After Filter) indicating similar quality between samples and mapped replicates.

### Search for enriched regions

**Fig 2:**
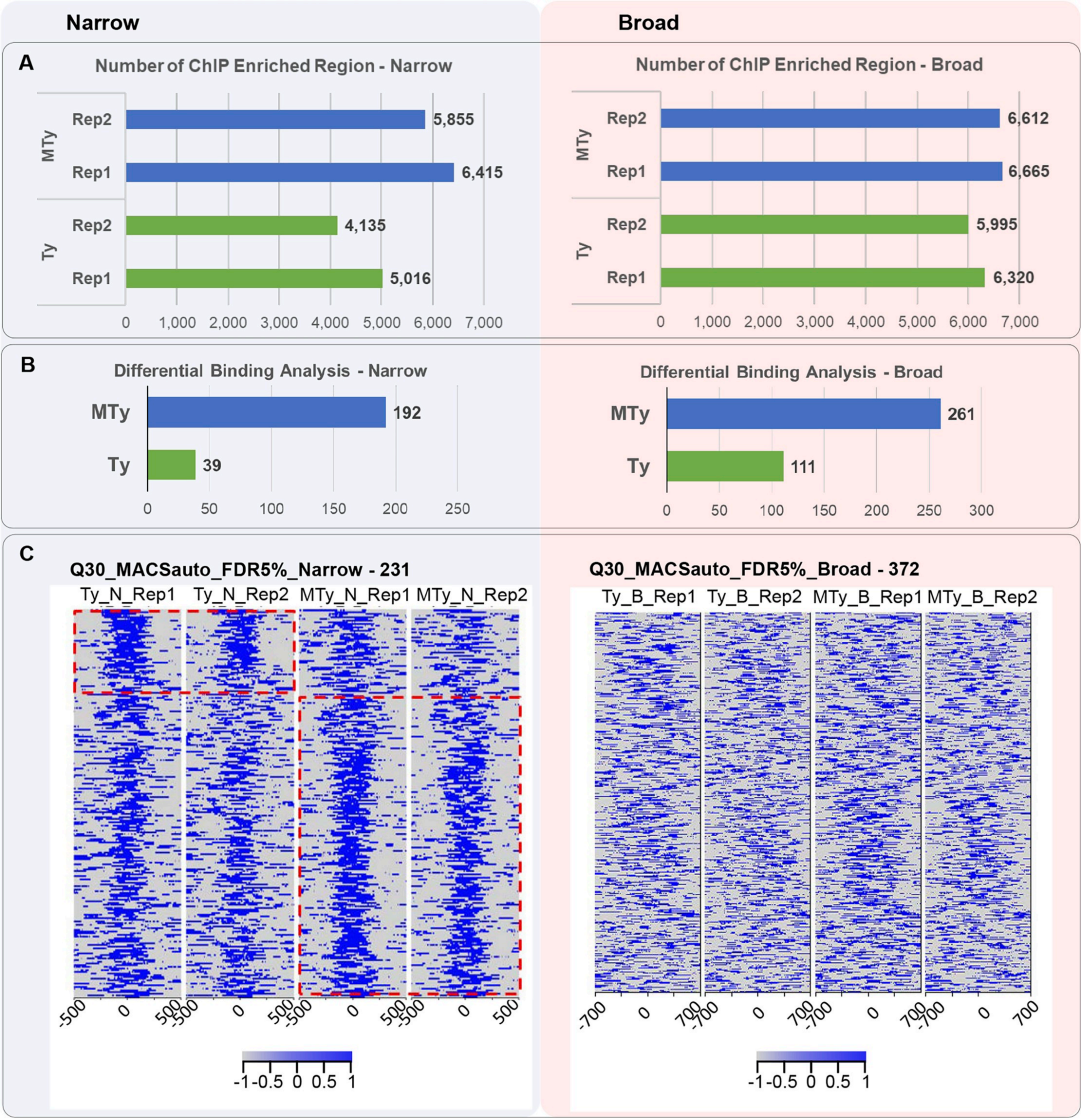
Peak calling and differential binding analysis. A—Barplots show the number of enriched peaks found by the MACS2 tool for each sample and replicate. The number of enriched regions is shown in each condition by the blue bars (MTy) or green bars (Ty). B–Differential binding analysis employing Narrow or Broad parameters, a threshold FDR 5% was applied. **C**—Enrichment profile of the differentially represented Narrow and Broad regions for MTy and Ty samples. Each profile contains four "heatmaps", one for each sample and match replicate (Narrow: Ty_N_Rep1, Ty_N_Rep2; MTy_N_Rep1, MTy_N_Rep2, Broad: Ty_B_Rep1, Ty_B_Rep2; MTy_B_Rep1, MTy_B_Rep2). Each horizontal line, ordered by fold-change from higher to lower values (top to bottom), depicts the enriched region, delimited in blue (gradient of color represents depths of reads). The predominantly enriched regions in MTy or Ty are delineated by the red dotted box, and are only noticeable in Narrow-parameter analysis. The total numbers of identified enriched regions using the specific parameters are shown in the title of the heatmaps. The enriched regions obtained by the stacking of reads were delimited from their center as -500 to 500 bp for Narrow and– 700 to 700 for Broad regions.

The search for enriched regions was performed independently in the samples using parameters to identify narrow (Narrow) or broad (Broad) regions, as described in Methods. The MACS2 program was configured to remove duplicated reads. The program used INPUT data from each sample as background control and the effective genome size. This way, using narrow parameters, a higher number of enriched regions in MTy (6415 for replicate 1 and 5855 for replicate 2) was detected when compared with Ty (5016 for replicate 1 and 4135 for replicate 2), considering 5% FDR (Fig 2A). Similarly, using broad parameters, a higher number of enriched regions was detected in MTy (6665 for replicate 1 and 6612 for replicate 2) comparatively to Ty (6320 for replicate 1 and 5995 for replicate 2). Tests for duplicated reads exclusion employing other MACS parameters led to the same general conclusion (S5A Fig).

The normalization and differential binding analysis of the regions enriched in MTy and Ty were performed using the program DiffBind. Independent analysis was performed for the enriched regions obtained using Narrow and Broad parameters (Fig 2B). A threshold of 5% FDR was used to define differently recognized regions showed in S3 Table. Using Narrow parameters, 192 regions were prevalent in MTy, in contrast to 39 in Ty. Alike, 261 regions in MTy and 111 regions in Ty were detected when Broad parameters were applied. Thus, a higher number of enriched regions prevalent in MTy were identified by Narrow and Broad analysis (Fig 2B). Modification of MACS and thresholds parameters led to the same results (S5B Fig).

The heatmap allows visualization of the differences of enriched regions, which represent possible sites of interaction of nitrated proteins with the chromatin, in MTy and Ty (Fig 2C) for Narrow or Broad analysis. Each horizontal line of the *heatmap* represents one of those regions shown in a descending order by Fold Change (FC) values. Highly enriched narrow regions in MTy replicates as compared to Ty or in Ty replicates compared to MTy were delimited by red dashed boxes in Fig 2C. Changes in the enrichment pattern between MTy and Ty samples were not so expressive for Broad as they were for Narrow analysis (Fig 2C). A low depth of reads over the broad enriched bound regions or a large number of punctual interactions of proteins along the extension of the broad region, may explain these results. The differential binding analysis indicates changes in the interaction profile of some nitrated proteins with chromatin after incubation of trypomastigotes with ECM, in particular when Narrow parameters were used to identify the enriched regions.

**Fig 3:**
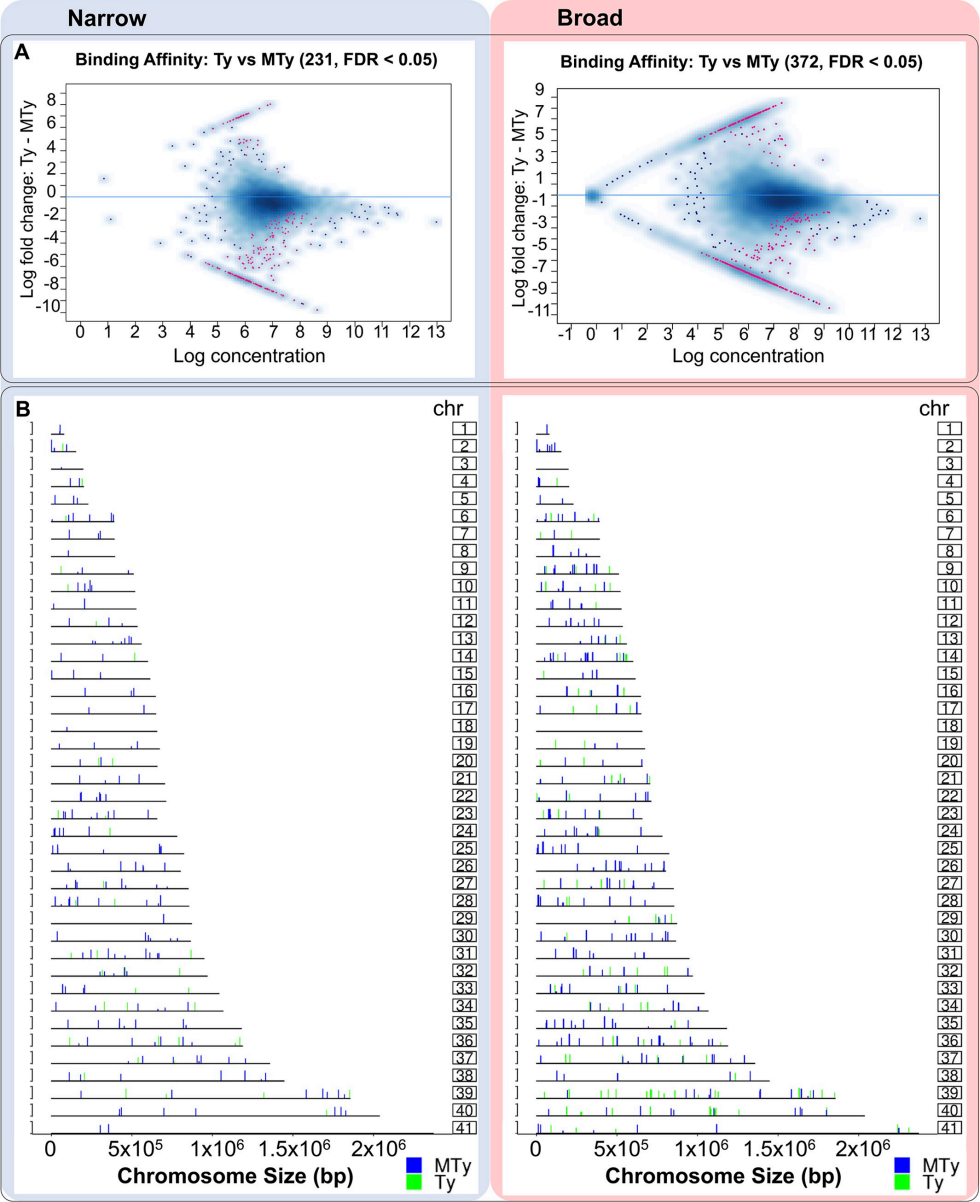
MAplot and localization of enriched regions throughout the Chromosomes. A—MAplots depict the enriched regions prevalent in MTy or Ty samples detected by MACSauto, with FDR 5% for Narrow (left) or Broad (right). The purple dots correspond to those regions enriched in MTy or Ty with FDR < 5%. B—Coverage plot representing the position of the Narrow (left) and Broad (right) enriched sequences obtained by MACSauto_FDR5%. The prevalent regions in MTy (blue bars) and in Ty (green bars) are represented throughout the 41 chromosomes (chr) of the *T*. *cruzi* CLBrener Esm-like haplotype. The height of each bar is relative to fold change values calculated by the DiffBind program.

A significant positive correlation (Sperman, two tailed, p <0.05) was found between the size of the chromosomes with the total number of enriched regions (ePks), differentially represented in MTy and Ty (S6 Fig—Left), as well as with the ePks differentially represented in MTy or in Ty, separately (S6 Fig—Right). The regions prevalent in MTy or Ty (Fig 3) are distributed along almost all chromosomes of the *T*. *cruzi* genome, with no hotspots for differential binding regions observed. A higher number of differentially represented narrow regions in MTy was identified in most of the chromosomes (e.g. 26, 35 and 38). For the Broad analysis, chromosomes 36, 37, 39 and 40 presented the largest number of regions identified in MTy and no differential regions were found on chromosomes 3 and 18. It is noticeable the larger number of binding regions in many chromosomes from MTy (e.g. 8, 9, 12, 14, 25, 26, 30, 31, 35 and 36) in comparison to Ty, in this particular case restricted to fewer chromosomes (e.g. 7 and 39) (Fig 3B- Broad). Although showing lower number of binding regions, a larger number in Narrow analysis was obtained in MTy (Fig 3B- Narrow). Of note, the distribution of the binding sites mapped along the chromosomes is also distinct when MTy and Ty are compared in Narrow and Broad analysis (Fig 3B). Although there was not a marked difference in reads density in broad regions enrichment between MTy and Ty samples as observed for narrow regions (Fig 2C), the presence of large genomic regions with marked FC values indicates that they also may be involved with parasite chromatin remodeling triggered by incubation with ECM.

Experimental evidence suggests that polycistronic transcription in *T*. *brucei* and *L*. *major* initiates at the divergent SSRs (Strand Switching Regions), with epigenetic factors playing a role [14,46,47]. Some components of ISWI complex involved with RNA Pol I and II transcription regulation in trypanosomatids [48] were also found enriched in divergent and convergent SSRs in *T*. *brucei* [49]. Besides, the genomes of *T*. *cruzi*, *T*. *brucei* and *L*. *major* show high synteny with approximately 6,200 conserved genes, 94% of which arranged on the same directional clusters [50]. An in house script was implemented to delineate the SSRs (Strand Switching Regions) on each *T*. *cruzi* chromosome and to check how many regions differentially enriched are located within the convergent or divergent annotated SSRs in MTy or Ty that have been identified by the ChIP-seq experiments. By this method, 386 convergent and divergent SSRs were found throughout the *T*. *cruzi* genome. No SSR was detected on chromosome 1, showing that all of its genes are transcribed from a single strand. From the 231 Narrow enriched regions, only two regions were found within SSR putative regions and from the 372 Broad enriched regions, 5 were found within convergent or divergent SSR, indicating that the interaction of nitrated proteins with *T*. *cruzi* DNA may not be playing a prominent role in the initiation of transcription.

### ChIP-Seq data quality

**Fig 4:**
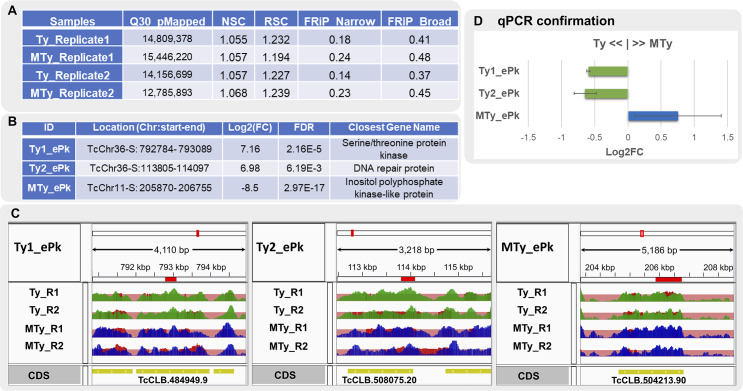
Quality metrics and validation of differentially bound regions described. A**–**Post-mapping cross correlation metrics (Normalized Strand Coefficient–NSC; Relative Strand Correlation—RSC) and Fraction of Reads in Peaks (FRiP) of both replicates of MTy and Ty samples using Narrow or Broad parameters. Q30 pMapped refers to the Phred score used. B–Enriched regions (ePks) selected for validation by qPCR. From left to right the columns correspond to: identification of sample origin, their coordinates location in the *T*. *cruzi* CL-Brener Esm-like genome, the value of log2 Fold Change (FC) and False Discovery Rate (FDR) calculated by the DiffBind and the identification of the nearest gene, respectively. C–Read coverage of the validated regions as depicted by IGV. Chromosomes are identified in the top row (IGV output). In the second row, samples are identified (MTy, blue and Ty, green) along with the length of the region in base pairs. The red rectangles indicate the zoomed area. In the third row, the treatment bedgraph files (pileup MACS output) are overlaid with respective lambda local control bedgraph files for each sample replicate represented by tones of red color in each track. The position and direction of the annotated CDSs are shown in yellow (lower row). D—Barplot representing alteration of regulatory regions by incubation of the parasite with ECM (MTy). The bars represent the values of the number of copies determined by log_2_(MTy/Ty) for each sample, after qPCR analysis. The blue bar corresponds to the enriched region identified preferentially in MTy and the green bar is the region identified preferentially in Ty. Regions confirmed by qPCR correspond to those presented in B. Standard deviations are presented by horizontal bars.

To evaluate ChIP-Seq data quality, strand specific cross-correlation (Normalized Strand Cross-correlation (NSC) and Relative Strand Cross-correlation (RSC) coefficients) and FRiP (Fraction of Reads in called Peaks) metrics were calculated for the MACSauto_FDR5% data (Fig 4A). For this work, the NSC (above 1.05) and RSC (above 1.1) values for each sample are in accordance with the recommendations (> 1.05 and >0.8, respectively) and the peaks referring to the average size of the fragments exceeded the average reads size (S7 Fig), an indicative of good data quality. Measure of the global enrichment was obtained by FRiP, using the coordinates of the enriched regions detected. Even though the values are in accordance with ENCODE recommendation (above 0.01), more pronounced FRiP values were obtained for Broad regions (above 0.37) compared with Narrow (above 0.14), though less visualization of enrichment was observed for the first. This is due to the large area occupied by these regions, and the possible low representativeness of reads, making it difficult to visualize the enrichment for these regions. Thus, the post-sequencing cross-correlation and FRiP metrics indicate good quality data, with a high signal rate compared to noises in the positions of the enriched regions found.

### Confirmation of enriched regions by qPCR

Primers were designed for 10 regions found by the pipeline (S1 Table) and three of them were confirmed by the qPCR technique. For 7 regions it was not possible to standardize the assay for an accurate evaluation. The qPCR from these regions presented high values of standard deviation between replicates or they presented a complex dissociation curve with more than one peak, indicating non-specificity in the amplification reaction. One enriched region differentially represented in MTy and two in Ty (Fig 4B) validated by qPCR, were in accordance with profiles from the ChIP-seq analysis (Fig 4C). Fig 4C shows the location, the closest neighboring regions and the coverage profile for each validated region in the sample replicates. The differences of coverage in MTy and Ty are noticeable after the normalization of local background. The differences observed in Fig 4D barplot were statistically significant between MTy and Ty samples using t-Student test analysis (p <0.05, two tail) from two biological replicates and three experimental replicates. The three regions, differentially represented in MTy or Ty were validated by independent techniques showing changes in the interaction profile of nitrated proteins with chromatin, as a consequence of the parasite incubation with ECM.

## Discussion

It is known that post-translational modifications on nuclear proteins interacting with genomic DNA alters chromatin status and are crucial to modify nuclear functional processes such as replication or transcription in different organisms. In addition, we have previously observed that exposure of trypomastigotes to ECM, an obligatory condition for host cells invasion, correlated with a differential nitration of histones [4].

Interaction of the infective form with ECM leads to a broad spectrum of post-translational modifications of proteins in *T*. *cruzi* trypomastigotes, including phosphorylation [3,21], nitration and S-nitrosylation [4]. In this case, modulation of protein nitration was attributed to the moderate Nitric oxide synthase activity described in the parasite, and consequently, may possess physiological role.

Here we show that trypomastigote incubated with ECM resulted in higher number of putative interaction sites of nitrated proteins with chromatin when compared to the control cells (Ty). We also identified Y29 nitrated residue in Histone 2B. The analysis of differential binding sites, comparing MTy with Ty, revealed no sequence-specific sites, nor a consistent association of nitrated-proteins binding sites with annotated divergent or convergent strand switch regions. Nevertheless, the binding sites were not randomly distributed along the chromosomes, conserving only a good correlation of number of sites with the length of the chromosome.

Interestingly, the parameters used for the analysis of differential binding sites (Narrow or Broad analysis) (Fig 2B), permit to extract different and complementary information. Narrow parameter analysis is frequently used to identify interaction of proteins with constricted regions in the genome, such as transcription factors binding features. We clearly observe, in this case, marked differences in the peaks of nitrated protein binding sites whether the parasites were exposed to ECM or not. In contrast, with the Broad parameters, the differences between peaks, in one or the other condition (MTy or Ty), are less expressive (Fig 2C).

Nevertheless, when we investigated the number and distribution of the nitrated proteins enriched binding sites along the chromosomes, the Broad analysis is more informative. It is noticeable, in this case, the high number of binding regions in many chromosomes of parasites exposed to ECM (for example chromosomes 8, 9, 12, 14, 25, 26, 30, 31 in MTy). Even the number of binding sites observable in the absence of ECM (Ty) is more numerous with the Broad analysis (for example chromosomes 7, 17 and 39) (Fig 2B and Fig 3B). The broad analysis is set to investigate interactions of proteins with DNA that happen as extended regions and it is useful to detect macromolecular complexes such as those that modify the status of chromatin [51]. Interestingly, we observed that histones are highly represented among the nitrated proteins that bind to genomic DNA, and also to a larger number of sites after exposure to ECM. This finding is compatible with the hypothesis that nitration of nuclear proteins, particularly histones, in response to ECM bind to specific genomic regions possibly modifying accessibility to DNA and therefore modulating activity of these regions.

The first obvious genomic region to investigate nitrated proteins binding sites would be the transcription initiation sites. It is known that epigenetic factors modulate access of the transcription machinery in the divergent SSR, the location of major transcription initiation activity of RNA polymerase II in trypanosomatids [47]. Therefore, all the divergent strand switch regions (dSSR) between two posttranscriptional units (PTUs) were identified and a possible correlation of nitrated proteins binding sites with the annotated dSSR in the *T*. *cruzi* genome was investigated. We found no correlation; only a few ChIP-seq enriched regions were detected within SSRs (2 and 5 for Narrow or Broad analysis, respectively).

If the data indicate no major contribution of the nitrated proteins with the *T*. *cruzi* main sites of transcription initiation, we must not rule out the role of the modified proteins in other regulatory layers of genetic activity that depends on chromatin assembly. The histone-DNA interactions when modified by PTMs may lead to altered chromatin assembly at the identified binding sites and may, epigenetically, affect gene expression [48]. In fact, one of the levels they could operate is elongation of transcription at discrete regions of the genome. This hypothesis finds support in studies conducted in *T*. *brucei* in which knockdown of a FACT (Facilitates Chromatin Transcription) complex subunit, shown to participate in RNA Pol II elongation in other organisms, led to significant loss of some histones in SSRs and within PTUs associated with a decrease in the levels of some RNA Pol II transcripts [52]. Different methylation levels of H3 K79 have been associated to cell cycle progression both in *T*. *cruzi* and *T*.*brucei* [11,53]. Other nuclear processes could somehow be affected by reorganization of the chromatin triggered by modified patterns of PTMs of DNA binding proteins (such as nitrated histones) after exposure of the parasite to the ECM.

Numerous histone modifications have been reported in trypanosomatids, in which their post-translational modifications are qualitatively and quantitatively different in replicative (epimastigote) and non-replicative (trypomastigote) life forms of *T*. *cruzi* [11,54]. Although protein tyrosine nitration was described in a number of species, from yeast to man, related to signaling mechanisms, nitration of histones is frequently associated with pathological conditions [55]. Herein we confirmed the nitration of Histones in response to the interaction of *T*. *cruzi* trypomastigotes with ECM [4] and identified the tyrosine residue modified in H2B histone (Y29) (Fig 1), a residue also modified by hydroxylation (Uniprot ID B5U6T3). *In vitro*, tyrosine nitration induces structural changes on H2B [56] and H3 histones [57], such as hyperchromicity, cross linking, loss of ß-sheet structure and α-helix of H2B, producing a partially folded structure in the native H2B [56], that may contribute with other PTMs to the modulation of their interaction with the neighborhood. In relation to other PTMs in histones it is well known in *T*. *brucei* that acetylation of histone H4 in K4 and K8 (80% and 10%, respectively) [53] is involved in the assembly and organization of chromatin [58], although the mechanism of action is unknown [56,57]. Acetylation of H4 K10 and K4 as well as other PTMs in Histones have been related to the control of gene expression and cell cycle regulation in *T*. *cruzi* [11,54,58,59]. PTMs in histones are also associated with the FACT activity [52]. This molecular machinery causes the necessary separation of the H2A and H2B dimers to allow passage of RNA polymerase, being critical for nucleosome organization during transcription [60]. Changes in H2B histones that intensify or attenuate their interaction with other members of the nucleosome complex could be critical for transcription control and/or chromatin rearrangement.

The interaction of nitrated proteins with specific genomic sites and the marked differences of the nitrated protein binding sites observed after exposure to ECM might act as a fine-tuning modulatory event, in critical regions of the genome relevant for signaling pathways necessary for the parasite adaptation to the impending new environment upon host cell invasion.

## Supporting information

S1 Fig *In silico* replicate pipeline. Flowchart of the multi-replicate pipeline. Red arrows indicate the independent processing of the data obtained by the MACS tool using Broad parameters; blue arrows represent the independent processing of the data obtained using Narrow parameters.
(TIF)

S2 Fig DNA fragmentation pattern and test of sequencing and mapping. **A**—Agarose gel (2%) electrophoresis of *T*. *cruzi* trypomastigotes (Ty, not incubated with ECM) fragments obtained by sonication using Covaris S2, after the 3-step cell lysis protocol. The sample was sonicated for 10 min. Standard 100 bp was applied on the right lane (P100bp). **B**–Evaluation of the mapping of reads in different genomes. A pilot ChIP protocol was done with two different samples (Ty and MTy) and the mapping test of the reads sequenced in the reference *T*. *cruzi* CLBrener haplotypes (Esmeraldo–Esm or NonEsmeraldo–NEsm), *T*. *cruzi* CLBrener contigs not assembled and mouse genome.
(TIF)

S3 Fig Alignment of *T*. *cruzi* Histones 2B. Seventeen sequences were recovered from Uniprot using the term “Histone H2B” under `Protein name [DE]`and “Trypanosoma cruzi” in `Organism[OS]`fields and aligned using Clustal Omega web tool. The image of alignment was created by Jalview tool. The H2B B5U6T3 is highlighted in grey and the residue tyrosine 29 (Y29) and the other tyrosine residues corresponding to other histones H2B found by alignment are highlighted in red. The higher intensity of the blue color corresponds to the higher identity among residues.
(TIF)

S4 Fig Quality of sequenced reads. **A**–BarPlot showing the total of reads per quality (Phred based quality score–“Q Score”). Each plot corresponds to one sequencing run of the same pool of samples. **B**–Multiqc BoxWhisker plot showing the average of quality of all sequenced reads per base (Phred score per base–Illumina/Sanger 1.9 encoding). Each green line represents the average of quality per base for each one of the eight samples. **C**–BoxWhisker plots of each sample. The blue line represents the average quality per base, showed as green line in B. The last sequenced base for each read was used as an extra cycle in sequencing to improve quality of the preview base and further trimmed. Only 75 cycles (150 cycles paired-end) were considered.
(TIF)

S5 Fig Peak Calling and Differential Analysis. **–A—**Barplot containing the enriched peaks found by the MACS2 tool from all the reads obtained after the Q30 mapping quality filter (Q30_MACSall) or by the selection of reads arranged in a binomial distribution model selected by the MACS2 program by the option "- auto-dup" (Q30_MACSauto). These two data sets were generated for parameters defined for narrow region search (Narrow–**A left**) and for defined parameters for broader region search (Broad–**A right**). Number of enriched regions found independently for Ty samples from both replicates are shown next to the green bars and for MTy samples, shown next to the blue bars. **B**—Barplot containing results of the comparative analysis of the enriched regions Narrow (**B left**) and Broad (**B right**) by the program DiffBind. Four results are shown in each graph, obtained from the enriched regions identified by the use of two parameter variations (MACSall, MACSauto), and by the restriction of differentially enriched regions between Ty and MTy by values of False Discovery Rate (FDR) less than 1 or 5%. The number of regions differentially found in Ty or MTy is at the right of the bars (Ty: green; MTy: blue).
(TIF)

S6 Fig Correlation between number of ePks and chromosome size. Correlation plot of the all narrow (**A—left**) or broad (**B—left**) enriched regions (ePks) differentially represented in MTy or Ty, and ePks divided into regions prevalent in Ty (green) or MTy (blue) versus the size of chromosomes of *T*. *cruzi* CL Brener Esm-like for narrow (**A—right**) or Broad (**B—right**) samples. The Narrow correlation coefficient was 0.47 for total ePks, 0.35 for Ty ePks and 0.43 for MTy ePks. All distributions showed significant statistical correlation with chromosome size (Sperman, two tailed, p <0.05): p-value Total < 0.0001, Ty = 0.0169, MTy = 0.0001. The Broad correlation coefficient was 0.69 for total ePks, 0.64 for Ty ePks and 0.50 for MTy ePks. All distributions showed significant statistical correlation with chromosome size (Sperman, two tailed, p < 0.05): p-value Total < 0.0001, Ty = 0.0002, MTy < 0.0001.
(TIF)

S7 Fig Cross-correlation profiles of the Ty and MTy samples (Replicates 1 and 2). The center of peak corresponding to read size consensus (75 bp) is represented by the dashed blue line and dashed red line corresponds to the center of the peak representing the average of fragment size for each sample (Ty-Rep1 145 bp, Ty-Rep2 140 bp, TyM-Rep1 155 bp, TyM-Rep2 135 bp).
(TIF)

S1 Table Primers Table—Primers constructed for validation by qPCR of the enriched regions differently found in Ty and MTy samples by *in silico* analysis. Primers of validated regions are in bold. (XLSX)

S2 Table Total proteins identified and quantified in the immunoprecipitated fraction combining the three conditions (IgG, Ty and MTy). The features reported for each protein ID are: protein IDs, protein description, number of proteins, peptides, mol. weight [kDa], score, normalized Intensity_IgG, normalized Intensity_Ty, normalized Intensity_MTy, normalized_iBAQ_IGG, normalized iBAQ_Ty and normalized iBAQ_MTy. Histones are marked in light orange. Protein contaminants are marked in grey.
(XLSX)

S3 Table Result of differential analysis of enriched regions identified in MTy and Ty samples. The columns show the differential region found and its closest gene. The information about the Peak is respectively the ID used internally (Internal ID), location of the peak (Chromosome name, start and end coordinates), width of the peak extension, attributes calculated by DiffBind (Concentration, Ty and MTy samples Concentration, Fold, p.value, FDR). DiffBind conc column show the mean read concentration over all the samples (the default calculation uses log2 normalized ChIP read counts with control read counts subtracted) and the mean concentration over the first (Ty) group and second (MTy) group. The Fold column shows the difference in mean concentrations between the two groups (Conc_Ty–Conc_MTy), with a positive value indicating increased binding affinity in the Ty group and a negative value indicating increased binding affinity in the MTy group. The final two columns give confidence measures for identifying these sites as differentially bound, with a raw p-value and a multiple testing corrected FDR in the final column. The information about the feature is its location (chrm, start, end, strand) in genome, disposal of the peak in relation of the feature, distance of peak from start of the feature, shortest distance of peak from feature, feature identification in TriTrypDB database and feature name. Each Excel sheet is concern to Narrow or Broad regions.
(XLSX)

S1 Text Chromatin Immunoprecipitation protocol. Detailed methodology based on Lee et al. 2006 (doi: 10.1038/nprot.2006.98) with modifications.
(DOCX)
