# Supplementary figures and images for: Global changes in nitration levels and DNA binding profile of Trypanosoma cruzi histones induced by incubation with host extracellular matrix
Source: PLoS Negl Trop Dis. 2020 May 29;14(5):e0008262. doi: 10.1371/journal.pntd.0008262 (PMC7286532; doi:10.1371/journal.pntd.0008262)

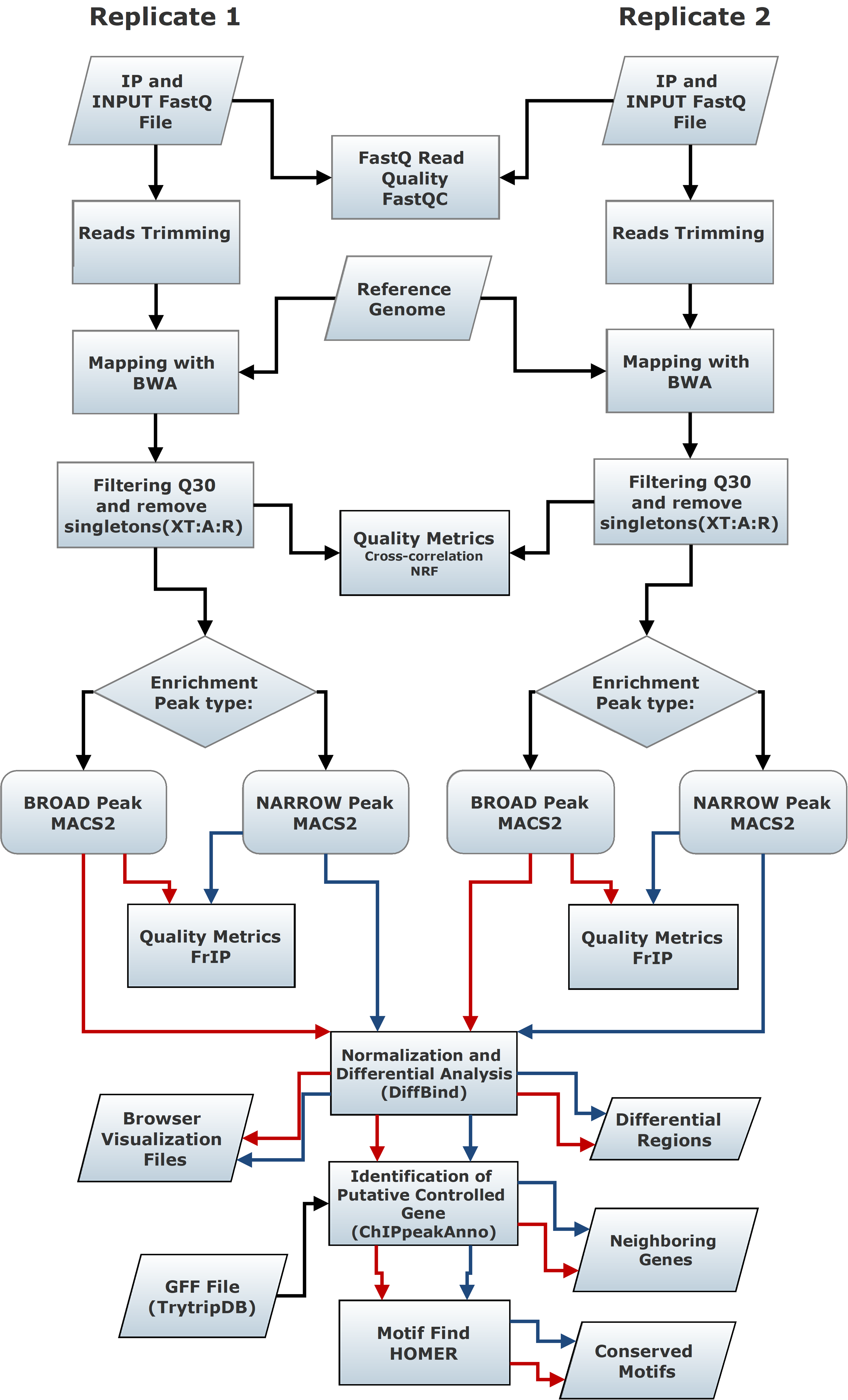

Supplement: S1 Fig — Flowchart of the multi-replicate pipeline. Red arrows indicate the independent processing of the data obtained by the MACS tool using Broad parameters; blue arrows represent the independent processing of the data obtained using Narrow parameters. (TIF) [file pntd.0008262.s001.tif]

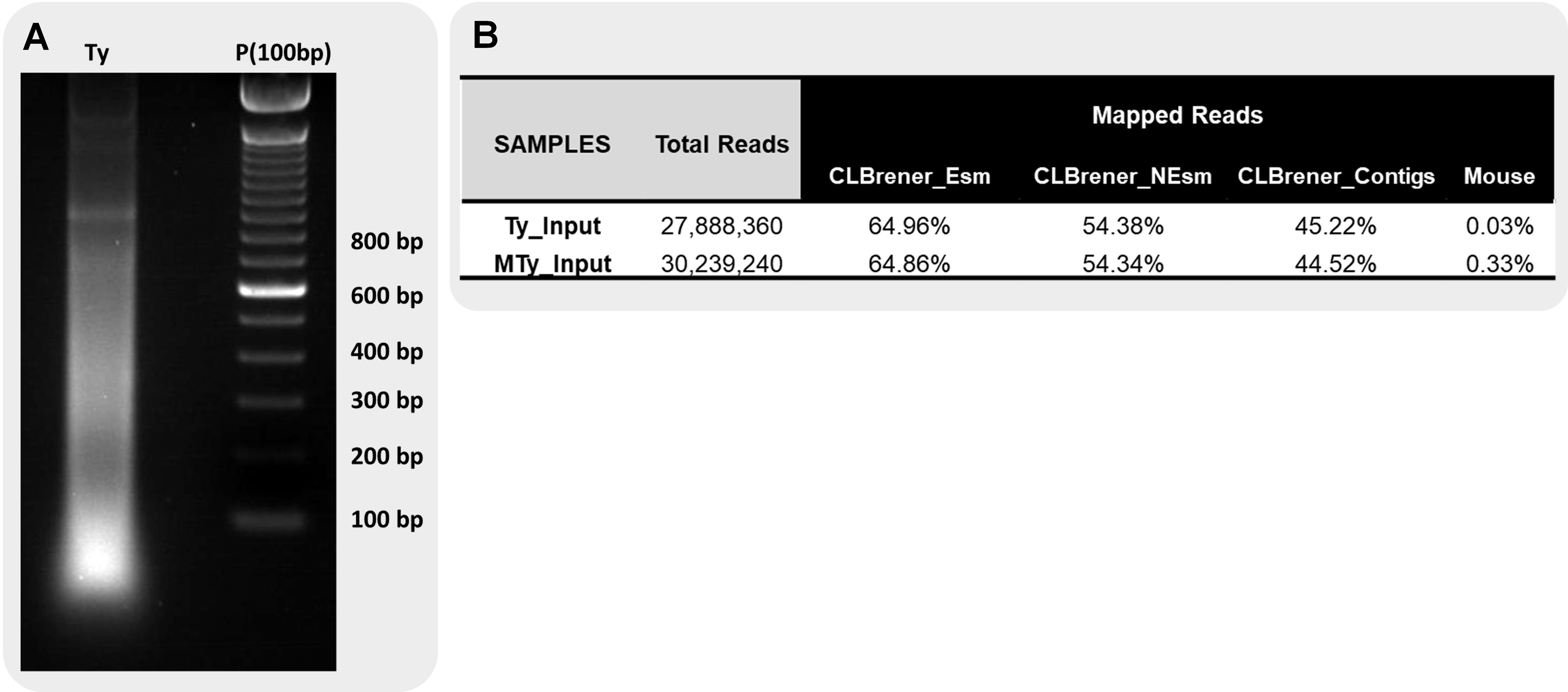

Supplement: S2 Fig — A—Agarose gel (2%) electrophoresis of T. cruzi trypomastigotes (Ty, not incubated with ECM) fragments obtained by sonication using Covaris S2, after the 3-step cell lysis protocol. The sample was sonicated for 10 min. Standard 100 bp was applied on the right lane (P100bp). B–Evaluation of the mapping of reads in different genomes. A pilot ChIP protocol was done with two different samples (Ty and MTy) and the mapping test of the reads sequenced in the reference T. cruzi CLBrener haplotypes (Esmeraldo–Esm or NonEsmeraldo–NEsm), T. cruzi CLBrener contigs not assembled and mouse genome. (TIF) [file pntd.0008262.s002.tif]

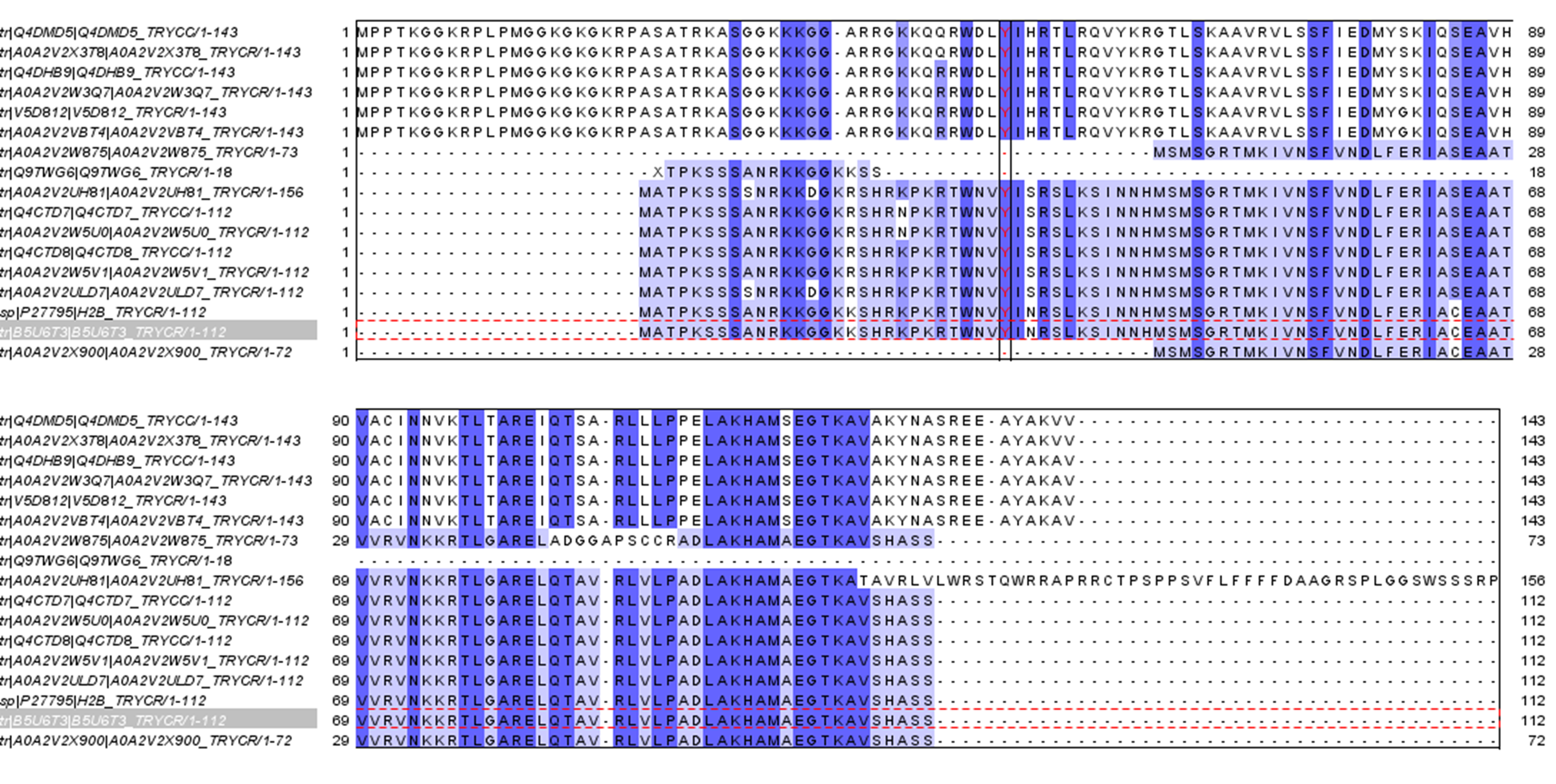

Supplement: S3 Fig — Seventeen sequences were recovered from Uniprot using the term “Histone H2B” under `Protein name [DE]`and “Trypanosoma cruzi” in `Organism[OS]`fields and aligned using Clustal Omega web tool. The image of alignment was created by Jalview tool. The H2B B5U6T3 is highlighted in grey and the residue tyrosine 29 (Y29) and the other tyrosine residues corresponding to other histones H2B found by alignment are highlighted in red. The higher intensity of the blue color corresponds to the higher identity among residues. (TIF) [file pntd.0008262.s003.tif]

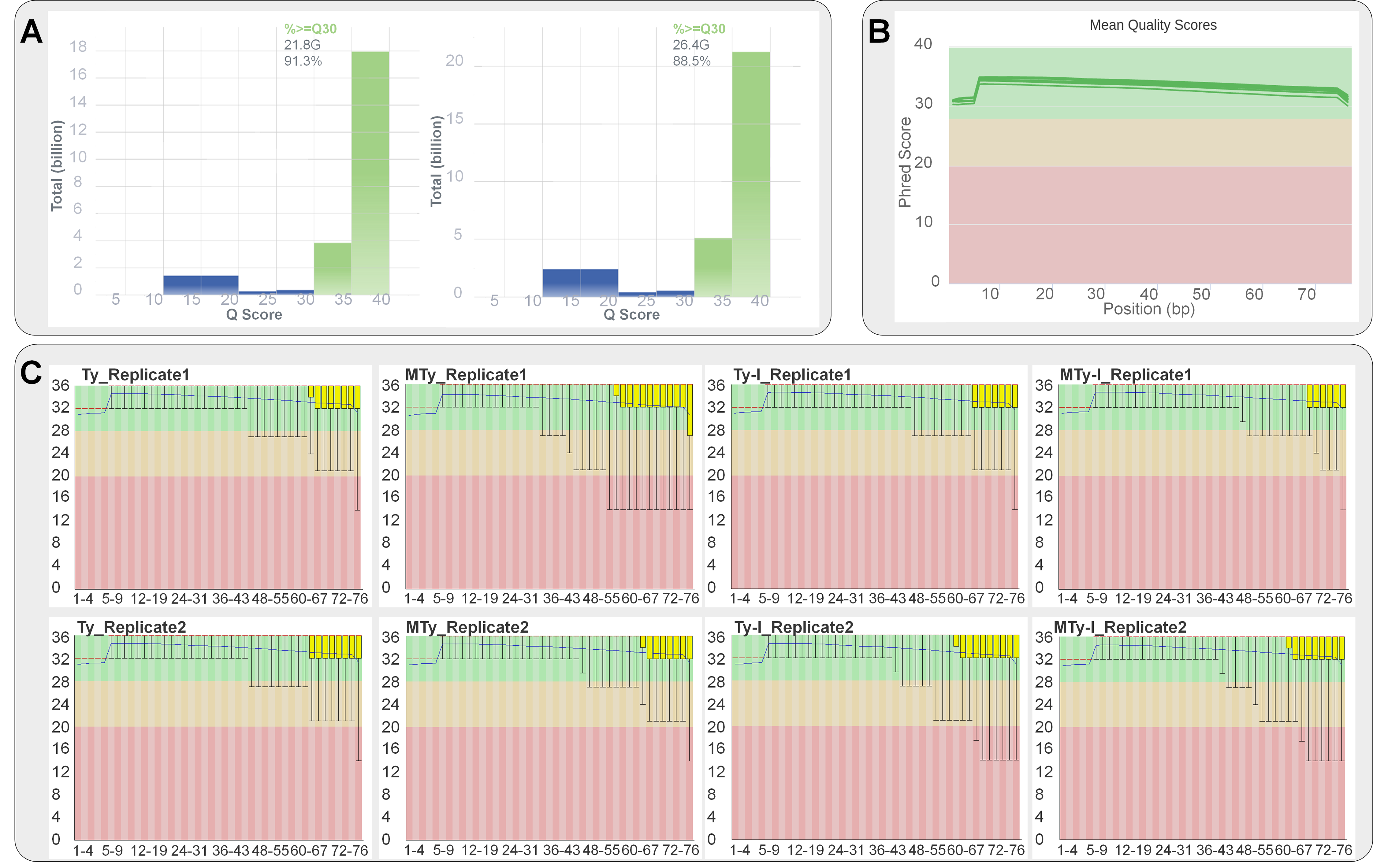

Supplement: S4 Fig — A–BarPlot showing the total of reads per quality (Phred based quality score–“Q Score”). Each plot corresponds to one sequencing run of the same pool of samples. B–Multiqc BoxWhisker plot showing the average of quality of all sequenced reads per base (Phred score per base–Illumina/Sanger 1.9 encoding). Each green line represents the average of quality per base for each one of the eight samples. C–BoxWhisker plots of each sample. The blue line represents the average quality per base, showed as green line in B. The last sequenced base for each read was used as an extra cycle in sequencing to improve quality of the preview base and further trimmed. Only 75 cycles (150 cycles paired-end) were considered. (TIF) [file pntd.0008262.s004.tif]

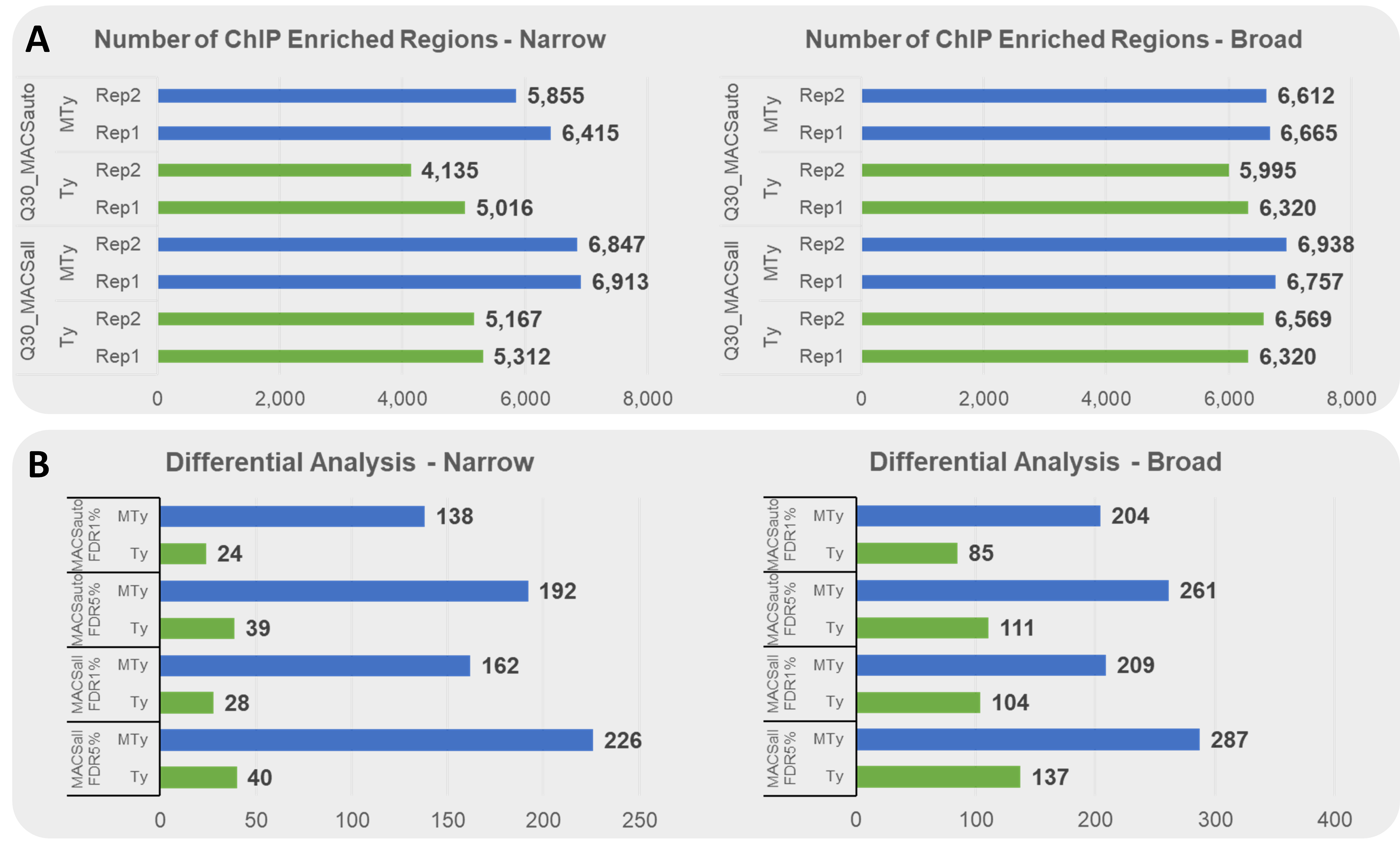

Supplement: S5 Fig — –A—Barplot containing the enriched peaks found by the MACS2 tool from all the reads obtained after the Q30 mapping quality filter (Q30_MACSall) or by the selection of reads arranged in a binomial distribution model selected by the MACS2 program by the option "- auto-dup" (Q30_MACSauto). These two data sets were generated for parameters defined for narrow region search (Narrow–A left) and for defined parameters for broader region search (Broad–A right). Number of enriched regions found independently for Ty samples from both replicates are shown next to the green bars and for MTy samples, shown next to the blue bars. B—Barplot containing results of the comparative analysis of the enriched regions Narrow (B left) and Broad (B right) by the program DiffBind. Four results are shown in each graph, obtained from the enriched regions identified by the use of two parameter variations (MACSall, MACSauto), and by the restriction of differentially enriched regions between Ty and MTy by values of False Discovery Rate (FDR) less than 1 or 5%. The number of regions differentially found in Ty or MTy is at the right of the bars (Ty: green; MTy: blue). (TIF) [file pntd.0008262.s005.tif]

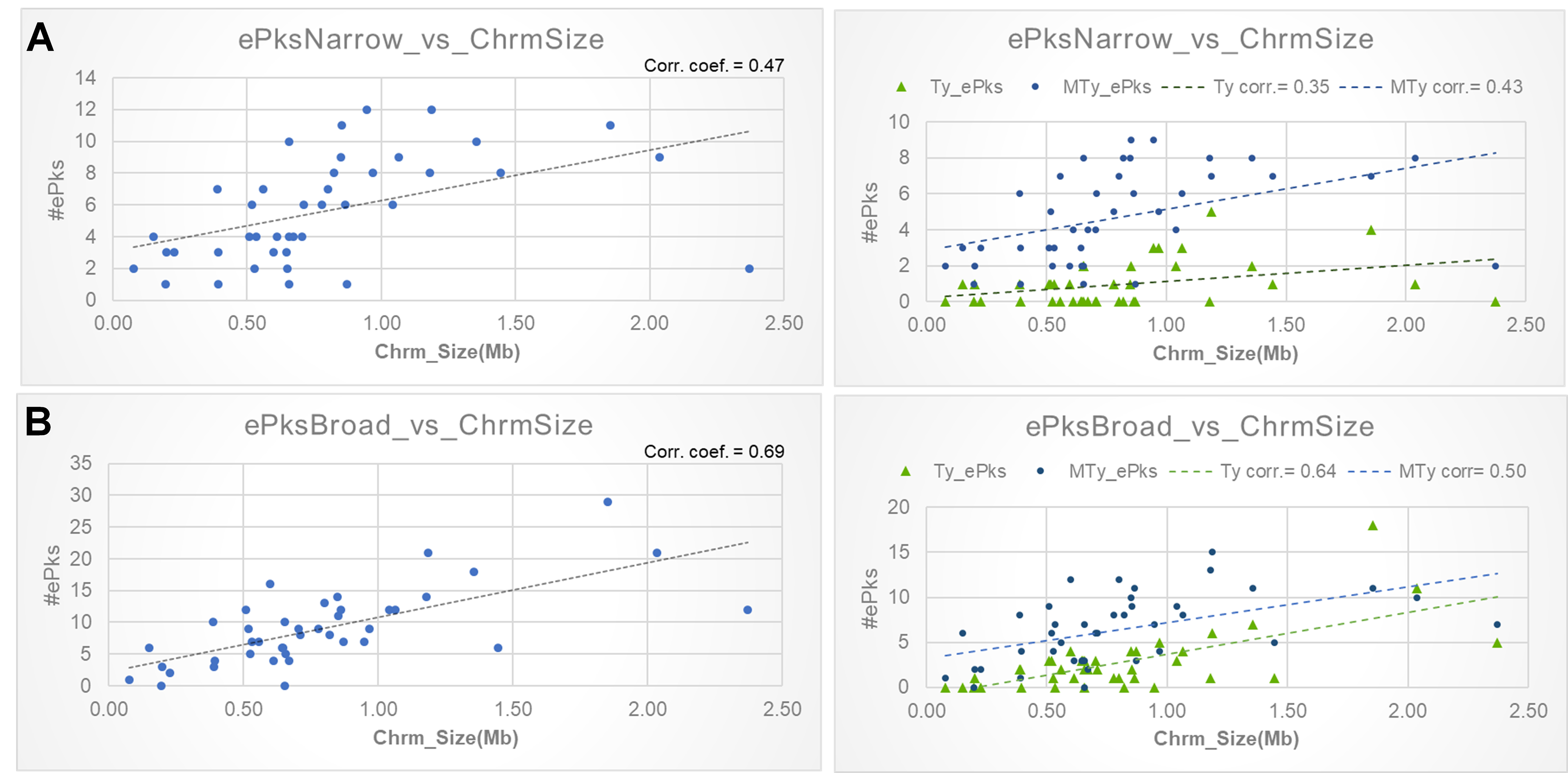

Supplement: S6 Fig — Correlation plot of the all narrow (A—left) or broad (B—left) enriched regions (ePks) differentially represented in MTy or Ty, and ePks divided into regions prevalent in Ty (green) or MTy (blue) versus the size of chromosomes of T. cruzi CL Brener Esm-like for narrow (A—right) or Broad (B—right) samples. The Narrow correlation coefficient was 0.47 for total ePks, 0.35 for Ty ePks and 0.43 for MTy ePks. All distributions showed significant statistical correlation with chromosome size (Sperman, two tailed, p <0.05): p-value Total < 0.0001, Ty = 0.0169, MTy = 0.0001. The Broad correlation coefficient was 0.69 for total ePks, 0.64 for Ty ePks and 0.50 for MTy ePks. All distributions showed significant statistical correlation with chromosome size (Sperman, two tailed, p < 0.05): p-value Total < 0.0001, Ty = 0.0002, MTy < 0.0001. (TIF) [file pntd.0008262.s006.tif]

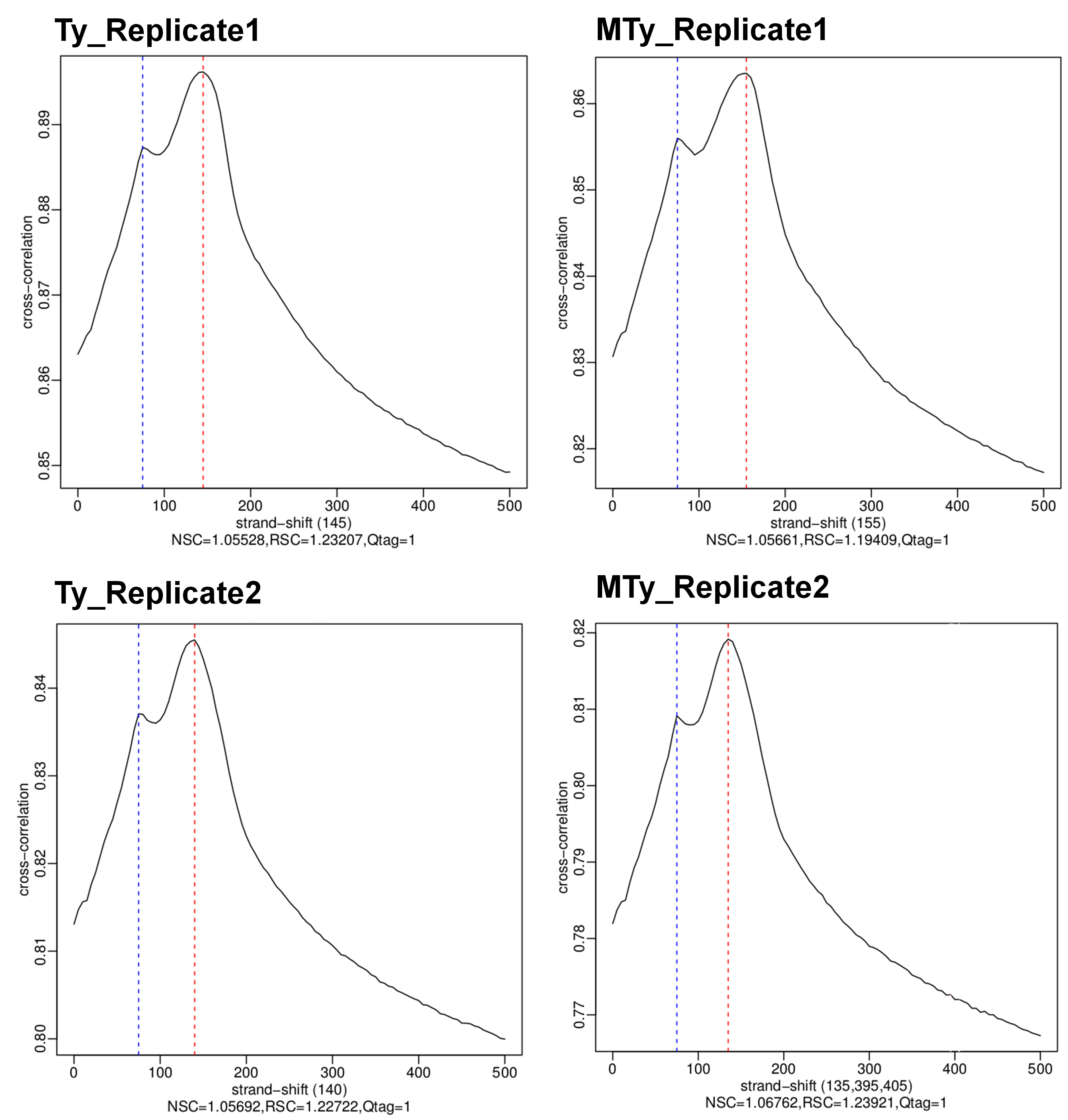

Supplement: S7 Fig — The center of peak corresponding to read size consensus (75 bp) is represented by the dashed blue line and dashed red line corresponds to the center of the peak representing the average of fragment size for each sample (Ty-Rep1 145 bp, Ty-Rep2 140 bp, TyM-Rep1 155 bp, TyM-Rep2 135 bp). (TIF) [file pntd.0008262.s007.tif]
