# Supplementary material for: Global changes in nitration levels and DNA binding profile of Trypanosoma cruzi histones induced by incubation with host extracellular matrix
Source: PLoS Negl Trop Dis. 2020 May 29;14(5):e0008262. doi: 10.1371/journal.pntd.0008262 (PMC7286532; doi:10.1371/journal.pntd.0008262)
Supplement: S1 Text — Detailed methodology based on Lee et al. 2006 (doi: 10.1038/nprot.2006.98) with modifications. (DOCX) [file pntd.0008262.s011.docx]

**S1 Text - Chromatin Immunoprecipitation protocol.** Detailed methodology based on Lee et al. 2006 (doi: 10.1038/nprot.2006.98) with modifications.

Trypomastigotes (5 x 10^8^ cells) from Ty e MTy samples were obtained as described in methodology and resuspended in 40 mL of PBS buffer with subsequent addition of 4 mL of formaldehyde solution (50 mM HEPES-KOH, pH 7.5, 100 mM NaCl, 1 mM EDTA, 5 mM EGTA, 3.3% Formaldehyde). After 20 min incubation, 2.5 mL of 2.0 M glycine (stock concentration) were added. The samples were centrifuged at 4,000 x g for 20 min. at 4°C and washed once with ice-cold PBS. The supernatant was discarded and the pellet incubated with 10 mL lysis buffer 1 (50 mM HEPES-KOH, pH 7.5, 140 mM NaCl, 1 mM EDTA, 10% glycerol, 0.5% NP-40, 0.25% Triton X-100, Protease and Phosphatase Inhibitors: SIGMAFAST Protease Inhibitor Tablets- SIGMA-ALDRICH, 0.1 mM PMSF, 0.1 mM NaF, 0.1 mM Na_3_VO_4_, 0.05 mM sodium β-glycerophosphate) for 10 min at 4°C under gentle agitation. After a second centrifugation round, the pellets were resuspended with 10 mL of lysis buffer 2 (10 mM Tris-HCl, pH 8.0, 200 mM NaCl, 1 mM EDTA, 0.5 mM EGTA, protease and phosphatase Inhibitors) and incubated for 10 min at room temperature under agitation. After a third centrifugation round, the new pellets were incubated with 2 ml lysis buffer 3 (10 mM Tris-HCl, pH 8.0, 100 mM NaCl, 1 mM EDTA, 0.5 mM EGTA, 0.1% sodium deoxycholate, 0.5% N-Lauryl sarcosine, protease and phosphatase Inhibitors), strongly vortexed and frozen at -80°C until next step. The sonication step was performed using Covaris S2 sonicator (Duty Cycle 5%, Intensity 4, Cycle per burst: 200, Continuous sonication: 10 minutes, Temperature Limit: 12°C). The sonication time to obtain size fragments between 100 and 800 bp was achieved after a standardization test (S1A Fig).

The entire volume of each sample was divided into two 1.5 mL tubes after addition of 220 μL of 10% Triton X-100 to 2 mL of the sonicated samples, followed by centrifugation (16,000 x g, 10 min, 4°C) and recovery of the supernatant. For each sample an independent 50 μL fraction named INPUT was collected and stored at 4°C for further use. To the remaining volume 0.9 mL of lysis buffer 3, 100 μl of 10% Triton X-100 and protease and phosphatases Inhibitors described above were added.

Prior to incubation with the sonicated sample, the beads (Dynabeads M-280 Sheep anti-Mouse IgG, NOVEX) were pre-blocked (washed three times with 1 mL of blocking solution: PBS containing 0.5% BSA), resuspended in 50 μL of blocking solution and incubated with anti-NO2Tyr antibody (Monoclonal anti-nitrotyrosine antibody, clone 1A6, Millipore; 100 μg magnetic beads to 5 μg antibody, overnight, 4°C, under stirring).

The sonicated samples were fractionated in two 2 mL tubes and 25 μL of pre-prepared solution containing magnetic beads and specific antibody in a final concentration of 150 mM NaCl were added to each tube.

The incubated samples were washed 5 times using 1 ml of RIPA buffer (50 mM HEPES, pH 7.5, 500 mM LiCl, 1 mM EDTA, 1% NP-40, 0.7% Sodium Deoxycholate) and once with 1 ml of TE buffer (10 mM Tris-HCl, 1 mM EDTA) plus 50 mM NaCl. Elution of the DNA-bound proteins was performed by incubating the sample with 200 μl of elution buffer (50 mM Tris-HCl, pH 8.0, 10 mM EDTA, 1% SDS) at 65°C for 60 min and vortexing every 15 min. After incubation, the samples were centrifuged (16,000 x g, 1 min. at room temperature) and the supernatant was recovered and transferred to a new tube. Elution buffer (150 μl) was added to the INPUT fractions and treated in the same manner as the immunoprecipitated samples from this step. For reverse crosslinking, the samples were incubated at 65°C overnight. The DNA was initially purified using enzymatic methods: incubation for 2 h at 37^o^C with 8 μL of RNase A (10 mg/mL), followed by incubation for 2 h at 55°C with 8 μL of Proteinase K (10 mg/mL) for each 200 μL of sample. Subsequently, the DNA was purified using QIAamp MinElute Virus Vacuum kit (QIAGEN) following the manufacturer's recommendations, with absence of RNA carriers in the buffer. The samples were quantified by Qubit Fluorimetric Quantification (Qubit dsDNA HS Assay Kit, Thermo Scientific) and used in the next protocol step, library construction and DNA sequencing described in the methodology.

Reference:

Lee, T. I., Johnstone, S. E., & Young, R. A. (2006). Chromatin immunoprecipitation and microarray-based analysis of protein location. *Nature Protocols*, *1*(2), 729–748. https://doi.org/10.1038/nprot.2006.98
